# Effect of heparin during extracorporeal detoxification in the severity of thrombocytopenia in patients with severe sepsis

**DOI:** 10.1186/cc11761

**Published:** 2012-11-14

**Authors:** VV Kulabukhov, AG Chizhov, AN Kudryavtsev

**Affiliations:** 1 Vishnevsky Institute of Surgery, Burn Center, Moscow, Russia

## Background

Disseminated intravascular coagulation plays an important role in the pathogenesis of severe sepsis. Current treatment of sepsis is not possible without the use of extracorporeal blood purification methods; the use of heparin is one of the measures of stabilization circuit. Intravenous administration of heparin can cause a decrease in platelet count. A reliable technique for differential diagnostics of heparin-induced thrombocytopenia is using 4T scales [1].

## Methods

An observational retrospective cohort study of 106 patients in the period from 2010 to 2011. The aim of the study was to determine the effect of heparin on the severity of clinical signs of heparin-induced thrombocytopenia in patients with severe sepsis. Inclusion criteria were the presence of severe sepsis in a patient (Surviving Sepsis Campaign 2008), the need for extracorporeal blood purification, and thrombocytopenia. The mean age of all the patients was 54 ± 15.91, mean SOFA score was 7.4 ± 0.9 (2 to 13). Reduced platelet counts were observed in 23 patients. Patients at high risk for heparin-induced thrombocytopenia type II (4 to 5 points for the 4T scale) in the study were not included. Ten of the patients (9.4%) conducted a session intermittent high-volume hemofiltration (group IHVH) within 4 hours, c volume replacement of 100 ml/kg/hour. Eleven patients (10.4%) did not receive extracorporeal blood purification (group Stand), because of uncontrolled bleeding. All patients received therapy according to Surviving Sepsis Campaign 2008. The standard dose of heparin was 8 to 15 units/kg/hour.

## Results

**Figure 1:**
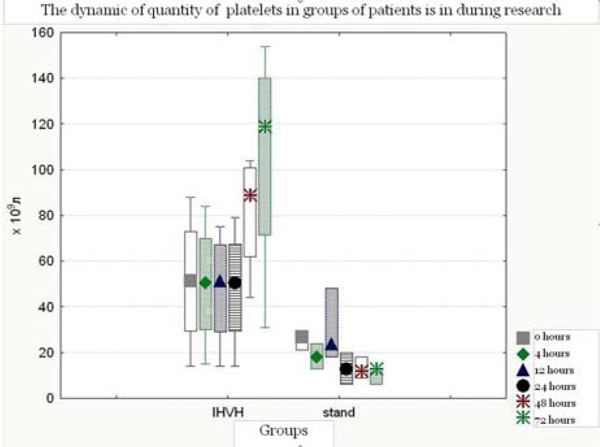
**Dynamics of platelets in the patient groups**.

In group IHVH the increase in platelet count at the end of the second day treatment was 105.75 ± 52.8 × 10^9^/l (Figure 1). This level is significantly different from the number of platelets in group Stand to the same phase of the study (10.7 ± 4.0 × 10^9^/l). During treatment in the groups studied there were no thromboses. The 28-day period mortality in group IHVH was 20% (two patients), and in group Stand was 9% (one patient).

## Conclusion

The use of heparin, including the extracorporeal blood purification, can be safe with heparin-induced thrombocytopenia type I in patients with severe sepsis.
